# The Relationship of the Subclavius Muscle with Relevance to Venous Cannulation below the Clavicle

**DOI:** 10.1155/2016/6249483

**Published:** 2016-02-07

**Authors:** Kyutaro Kawagishi, Joho Tokumine, Alan Kawarai Lefor

**Affiliations:** ^1^Department of Anatomy, Shinshu University School of Medicine, 3-1-1 Asahi, Matsumoto-shi, Nagano-ken 390-8621, Japan; ^2^Department of Anesthesiology, Kyorin University School of Medicine, 6-20-2 Shinkawa, Mitaka-shi, Tokyo 181-8611, Japan; ^3^Department of Surgery, Jichi Medical University, Tochigi 329-0498, Japan

## Abstract

*Introduction*. The catheter “pinch-off syndrome” has been described to be secondary to crimping of the catheter between the clavicle and the first rib, as well as entrapment of the catheter at the site of penetration of the subclavius muscle. The lateral insertion technique has been recommended to prevent catheter pinch-off, but it is unknown if this technique can prevent entrapment by the subclavius muscle. We undertook this study to evaluate the anatomical relationship of the subclavius muscle and the subclavian vein.* Methods*. Twenty-eight adult cadavers were studied on both right and left sides. The adherence between the subclavian vein and subclavius muscle was subjectively assessed and the distance between the two structures was measured in mm.* Results*. The subclavius muscle and subclavian vein were tightly adherent in 72% of specimens, partly adherent in 14% with a mean distance of 4.5 mm and loosely connected in 14% with a mean distance of 6.1 mm.* Conclusions*. The anatomical relationship between the subclavius muscle and vein was very close in the majority of specimens, suggesting that the lateral insertion technique may not prevent penetration of the muscle, which may contribute to catheter pinch-off. The real-time ultrasound-guided technique may prevent penetration of the subclavius muscle.

## 1. Introduction

Catheter compression by the clavicle and the first rib at the narrow costoclavicular space has been referred to as the “pinch-off syndrome” [1, 2]. Catheter pinch-off may cause resistance during infusion of intravenous fluid, difficulty drawing blood samples, or catheter fracture. Lateral insertion of the catheter into the subclavian vein is recommended to avoid catheter pinch-off, where the needle penetrates the vein in the wider space between the clavicle and the first rib avoiding occlusion of the catheter by bony structures [1].

In 1993, Magney et al. hypothesized that entrapment of the catheter by soft tissues, namely, the costoclavicular ligament and subclavius muscle, is another mechanism leading to catheter pinch-off [3]. Krutchen et al. revealed that entrapment by the subclavius muscle caused subclavian venous catheter crimping similar to the pinch-off syndrome (POS) without involving the bony structures [4]. Several investigators have recommended lateral insertion of the catheter to prevent the POS [5–9]. However catheter fracture has still been reported [10, 11].

We undertook this study to quantitatively evaluate the anatomical relationship between the subclavius muscle and the subclavian vein with regard to soft tissue entrapment. The goal is to determine whether subclavian vein catheterization can be performed without penetrating the subclavius muscle using the lateral insertion technique.

## 2. Materials and Methods

In this study, 56 veins in 28 adult Japanese cadavers (11 male cadavers, age 82–94, and 17 female cadavers, age 76–99) were used. All donors agreed to donate their bodies for medical education and research use by their living will. This study was approved by the medical ethics committee.

### 2.1. Anatomical Dissection

The skin and the subcutaneous tissue were dissected to identify the pectoralis major muscle, and the muscle was transected at its origin and reflected to expose the pectoralis minor muscle. The clavipectoral fascia (also known as the costocoracoid membrane or coracoclavicular fascia), which covers the area between the pectoralis minor muscle and the subclavius muscle, was then identified. The pectoralis minor muscle was transected at its origin to reveal the brachial plexus, the axillary artery, and the axillary vein. These structures were exposed by careful dissection, and the clavipectoral fascia was examined to determine the degree of connectivity between the subclavian vein and the clavipectoral fascia. Only loose adipocytes were removed and the tight connective tissue remained intact. The first author (Kyutaro Kawagishi) performed the procedures in all cadavers.

### 2.2. Measurements

The distance between the subclavian vein and the clavipectoral fascia was measured manually in the same manner in all specimens.

### 2.3. Statistical Analysis

The measured distances between the subclavian vein and the subclavius muscle in each group were compared and characterized with the Kruskal-Wallis test and Bonferroni correction. Laterality of the connectivity between the subclavian vein and the subclavius muscle was evaluated with Chi-square analysis. The distance between the subclavian vein and the subclavius muscle was analyzed with the Mann-Whitney *U* test. Laterality of the connectivity as well as the distance in cadavers' gender was compared using Chi-square analysis and the Mann-Whitney *U* test, respectively.

## 3. Results

**Figure 1:**
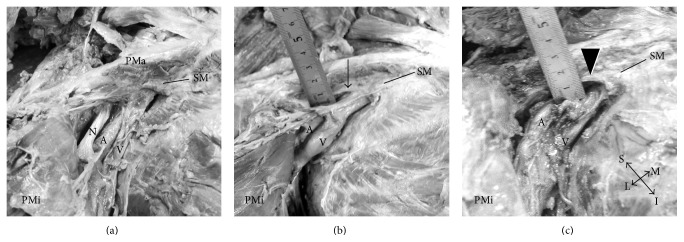
Anatomical relationship of the subclavian vein and the subclavian muscle. (a) Connected group. The subclavian vein (V) is tightly adherent to the subclavius muscle (SM) via the clavipectoral fascia. (b) Partly connected group. The lateral part of the subclavius muscle is not adherent to the subclavian vein (arrow). (c) Unconnected group. The subclavian vein is completely separate from the subclavius muscle (arrowhead), both laterally and medially. The space between the muscle and vein contains loose connective tissue composed of sparse fibers and adipocytes. PMa: pectoralis major (transected), PMi: pectoralis minor (turned over).

Figure 1 shows examples of three levels of connectivity between the subclavian vein and subclavius muscle via the clavipectoral fascia, which was divided into three categories, including the connected group (Figure 1(a)), the partly connected group (Figure 1(b)), and the unconnected group (Figure 1(c)).

In the connected group, the subclavian vein was tightly adherent to the subclavius muscle via the clavipectoral fascia (Figure 1(a)) and was seen in 40/56 specimens (72%). In the partly connected group, a tight connection to the clavipectoral fascia was limited to the medial part of the subclavian vein and the lateral part is loosely covered by sparse fibers with space-occupying adipose tissue (Figure 1(b)), which was observed in 8 veins (14%). The distance between the subclavian vein and the clavipectoral fascia in the partially unconnected specimens ranged from 2 to 8 mm (mean 4.5 mm). The unconnected group was loosely covered by the adipose tissues with sparse fibers, which easily removed (Figure 1(c)). This was observed in 8 veins (14%), and the distances ranged from 3 to 10 mm (mean 6.1 mm).

**Figure 2:**
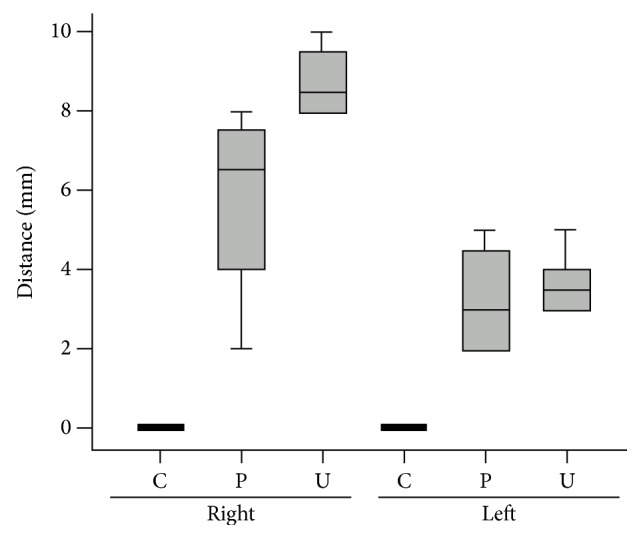
Distance between the subclavius muscle and the subclavian vein in each group on the right and left sides. A significant difference was found comparing the distance from the subclavius muscle to the subclavian vein among the three groups based on the subjective classification. C: tightly connected group, P: partly connected group, and U: unconnected group.

There was no correlation between the degree of connection between the vein and muscle based on gender (*p* > 0.05).  Figure 2 shows the correlation between the distances between the subclavian vein and the subclavius muscle in each group on each side. There was no significant difference in the degree of connectivity based on the side (right versus left) in the specimens evaluated for either the subjective determination of connectivity or the measured connectivity, in mm (*p* > 0.05).

## 4. Discussion

Based on these results, we determined that the subclavius muscle and subclavian vein are closely adherent in the vast majority of specimens evaluated, with 86% having tightly or partly connected veins and muscles. The anatomical landmark technique of subclavian venipuncture is usually performed, where “the needle is passed beneath the clavicle, with the needle hugging the inferior surface of the clavicle [12].” Based on that popular technique, prevention of penetration of the subclavius muscle during the venipuncture is difficult, because of the close anatomical relationship of the vein and muscle in the majority of specimens, as observed in this study.

Magney et al. recommend using the operator's thumb pressing the skin to make a deeper puncture point and a shallower angle for the needle [13]. This technique is likely to allow the catheter to avoid penetrating the subclavius muscle, if the subclavian vein is far from the subclavius muscle. To prevent the POS, the skin puncture site for the catheter using the usual landmark technique has been modified to be lateral to the midclavicular line [10]. This modification achieves a shallower needle-insertion angle but might not prevent penetration of the subclavian muscle, since the vein and the muscle are so closely adherent in a majority of specimens.

One possible limitation of this study is a difference in the extent of connectivity comparing a live patient and a cadaver. However, the adherence may in fact be less in a cadaver than in the living state with distension due to circulation, which would make the subclavian vein and the subclavius muscle even more adherent in the live human. One unexplained finding in this study was the variability observed in the fibrous tissue between the subclavius muscle and the subclavian vein.

The subclavius muscle is not a strong tissue compared to the costoclavicular ligament, which is present at the narrow costoclavicular space composed of the clavicle and first rib. The costoclavicular ligament is thought to have an additional role in the development of POS to entrap the catheter between the bony structures. The fascia covering the subclavius muscle is known as the clavipectoral fascia. This is a tight fascia situated between the pectoralis minor muscle and the subclavius muscle, which covers the axillary vessels and nerves. Traced upward, it splits to enclose the subclavius muscle and is also attached to the first rib medial to the origin of the subclavius muscle. Laterally, the clavipectoral fascia is thick and dense and is attached to the coracoid process. The portion extending from the first rib to the coracoid process is often denser than the rest and is sometimes called the costocoracoid ligament. With those anatomical definitions, it may be inferred that the anatomical cause of entrapment of a subclavian vein catheter might not be due to the subclavius muscle itself, but by the clavipectoral fascia.

**Figure 3:**
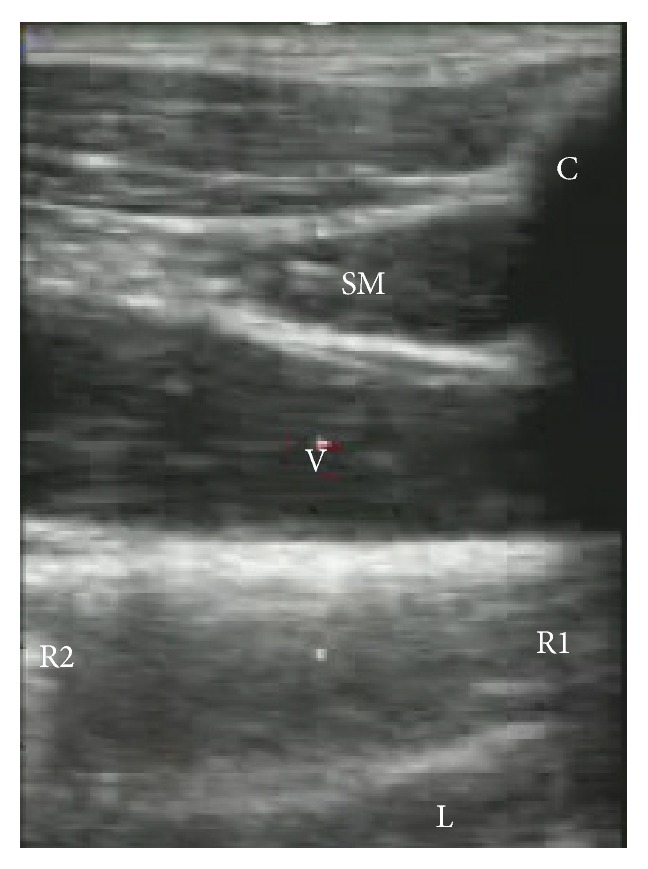
Ultrasound view of the subclavius muscle. SM: subclavius muscle, C: clavicle, V: subclavian vein and/or infraclavicular axillary vein, R1: first rib, R2: second rib, and L: lung.

Recently, ultrasound guidance [14–18] for central venipuncture has been recommended based on its clinical efficacy. The use of real-time ultrasound-guided catheter insertion may avoid penetration of the subclavius muscle, although further study is needed (Figure 3).

## 5. Conclusions

We conclude that use of the lateral insertion technique for subclavian venous catheterization using the anatomical landmark technique may make it difficult to avoid penetration of the subclavius muscle by the needle. The authors recommend use of real-time ultrasound guidance to avoid penetration of the subclavius muscle during vein catheterization based on clinical experience, which may help avoid the problem of catheter fracture due to entrapment. However, further studies are needed to evaluate the utility of ultrasound guidance to avoid penetration of the subclavius muscle.
